# Examining standardized tools used for the evaluation of mobile health applications for cardiovascular disease

**DOI:** 10.3389/fpubh.2023.1155433

**Published:** 2023-06-14

**Authors:** Jennifer Kircher, Walter Swoboda, Felix Holl

**Affiliations:** ^1^DigiHealth Institute, Neu-Ulm University of Applied Sciences, Neu-Ulm, Germany; ^2^Institute for Medical Information Processing, Biometry, and Epidemiology, Ludwig Maximilian University of Munich, Munich, Germany

**Keywords:** mHealth, mobile application, cardiovascular disease, evaluation, assessment, methods

## Abstract

Cardiovascular disease is one of the leading causes of death worldwide. Scarce resources and rising costs are pushing healthcare systems to their limits. There is an urgency to develop, optimize and evaluate technologies that provide more effective care for patients. Modern technologies, such as mobile health (mHealth) applications, can provide relief as a key strategy. To integrate digital interventions into care structures, a detailed impact assessment of all professional mHealth applications is needed. The aim of this study is to analyze the standardized tools used in the field of cardiovascular disease. The results show that questionnaires, usage logs, and key indicators are predominantly used. Although the identified mHealth interventions are specific to cardiovascular disease and thus require particular questions to evaluate apps, the user readiness, usability, or quality of life criteria are non-specific. Therefore, the results contribute to understanding how different mHealth interventions can be assessed, categorized, evaluated, and accepted.

## 1. Introduction

Studies such as *Neumann* et al. (1) have examined the growing number of digital health offerings, including mobile health (mHealth) apps, and expect them to double by 2025. Given the rapidly evolving market for digital health, there is an urgency to evaluate professional mHealth applications for their impact assessments. Such assessments will help determine whether the data and treatment outcomes are valid enough to provide quality care (2). Currently, there is no international consensus on standards for assessing health apps. Existing evaluation frameworks, such as the American Psychiatric Association app evaluation model, stand out in their flexibility of approach. However, this has also led to a demand for a more applied approach that provides more concrete information to users (3). An evaluation framework that identifies and instrumentalizes various exemplary methods and tools is lacking.

According to *Kvedar* et al. (4), developing, optimizing, and evaluating technologies that provide more effective care for patients is needed. mHealth interventions have great potential to support the treatment of cardiovascular diseases (CVD), which presents a considerable burden of diseases globally. In Germany, for example, 331,211 deaths were related to diseases of the cardiovascular system in 2019 (5). Earlier statistics from 2015 put the highest disease-specific illness costs in the German healthcare system at around 46.4 billion euros, caused by cardiovascular diseases (6). mHealth has the potential to improve CVD treatment by providing more personalized and timely care, supporting patient self-management, and encouraging healthy behaviors. Elements that mHealth applications can support are remote monitoring, patient education and self-management, as well as behavior change (7–9). However, developing applications for CVD comes with some unique challenges and obstacles compared to other disease-related apps. CVD is a complex condition that encompasses a range of different diseases and risk factors. Developing an app that addresses all aspects of CVD, from prevention to diagnosis to treatment, can be challenging (10). There is also a great variability in patient needs, as patients with CVD can have different needs and preferences depending on their specific condition and individual circumstances. Developing an app that is personalized and adaptable to different patient needs can be difficult (11).

The aim of this study is to identify which standardized tools are already used today for a comprehensive evaluation of mHealth applications in the field of cardiovascular disease. The resulting assessment, categorization, and evaluation findings will inform the development of an applicable evaluation framework for CVD mHealth interventions as a recommended course of action.

## 2. Materials and methods

To generate an initial impression of existing evaluation methods for assessing mHealth applications, a preliminary study has been conducted as part of a scoping review for CVD. The study “methods used to evaluate mHealth applications for cardiovascular disease: a quasi-systematic scoping review” (12) was published in the International Journal of Environmental Research and Public Health in 2021. The 38 studies already identified were narrowed down for the present study based on further exclusion criteria, and 37 studies (13–49) formed the starting point for this study. While the initial study provided an overview of all evaluation tools used, this study specifically looks at standardized tools that have been used in the evaluation of CVD mHealth applications and investigates their characteristics and possible shortcomings. Inclusion and exclusion criteria, the search strategy, and literature selection (including a table with the extracted data) can be found in the Supplementary material. These steps were done following the preferred reporting items for systematic reviews and meta-analyses (PRISMA) scheme (50).

We categorized the identified tools into 8 application areas and filtered by tool type. We derived the categories through thematic analysis (51). In addition, we subdivided the mHealth application according to their intervention type. Treatments performed only by using an app belong to the “mHealth app” type. When multiple devices or technical components, such as an app, an ECG (electrocardiogram), or a smartwatch, are used, the studies fall into the “mHealth system” group. Under “mHealth text messaging” those studies are assigned whose intervention is based exclusively on text messages, in particular “short message service”(SMS)-based messages. Following the categorization, we investigated the characteristics and shortcomings of the tools.

## 3. Results

### 3.1. Characteristics

**Figure 1:**
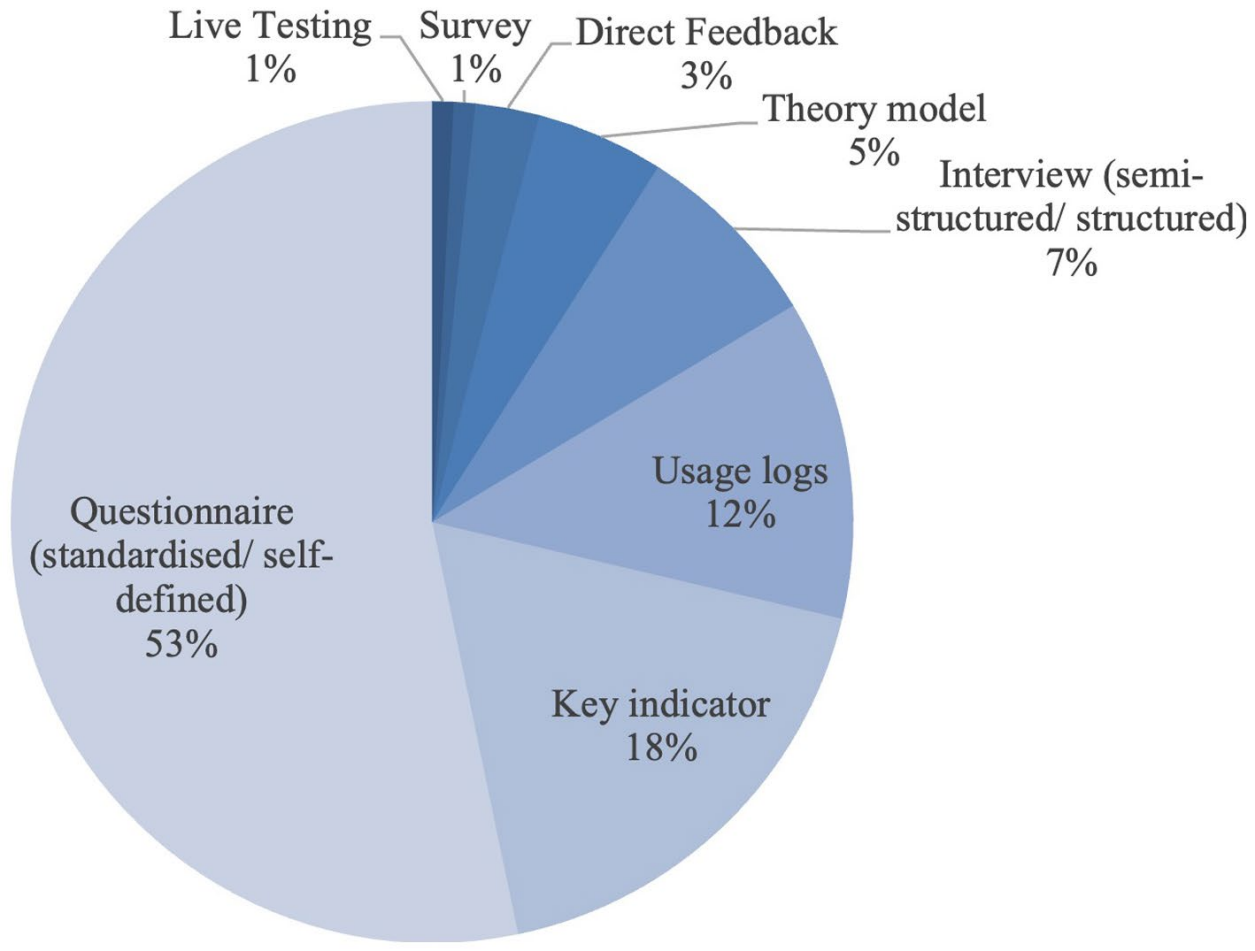
Instruments that were used most frequently.

Fourthy-eight evaluation instruments have been identified from the 37 studies. Of the 48 methods, questionnaires (*n* = 29, 60%) and economic measures such as key performance indicators (*n* = 9, 18%) are predominantly used as evaluation tools. All instruments have been used a total of 122 times. Besides questionnaires (*n* = 65, 53%) and key indicators, usage logs are used to assess user loyalty (*n* = 15, 12%) (Figure 1).

**Figure 2:**
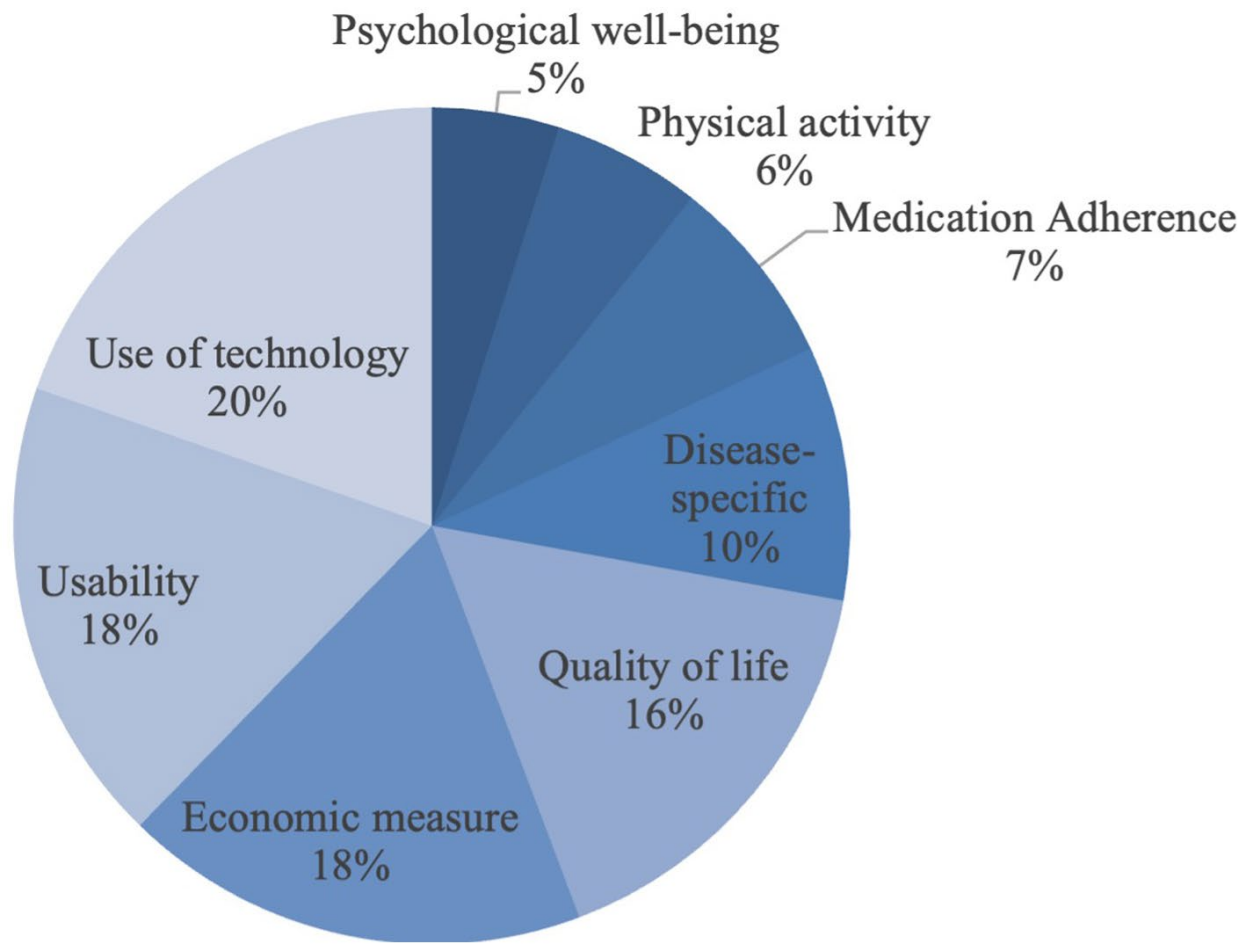
Dimensions that were used most frequently.

The dimension most frequently examined in all studies is the Use of Technology (*n* = 24, 20%). This examination is mainly carried out by usage logins (*n* = 15, 63%) and according to the theoretical construct of the “unified theory of acceptance and use of technology 2 (UTAUT2)” (*n* = 5, 21%). In addition, other important dimensions are usability (*n* = 22, 18%), quality of life (*n* = 20, 16%), and other economic measures (*n* = 22, 18%). The least used methods are those related exclusively to psychological well-being. Figure 2 shows the percentage of the 122 assessments conducted that apply to each dimension.

Among the 37 studies identified, just over half (*n* = 19, 51%) are mHealth systems, about a third are mHealth apps (*n* = 12, 32%), and 6 are applications for text messages. If the frequency of the implementation of the instruments is considered (Table 1), for mHealth systems (*n* = 63, 100%), both key indicators (*n* = 11, 17%) and usage logs (*n* = 10, 16%) are used almost equally. mHealth apps (*n* = 43, 100%) and mHealth text messaging (*n* = 16, 100%) are predominantly assessed by questionnaires.

**Table 1:** Type of intervention and the frequency of instruments.

Type of intervention and Evaluation instrument	Number of type of intervention
**mHealth system**	**63**
Interview	3
Key performance indicator	11
Model	4
Questionnaire	34
Survey	1
Usage logs	10
**mHealth app**	**43**
Direct feedback	1
Interview	5
Key performance indicator	7
Live testing	1
Model	1
Questionnaire	24
Usage logs	4
**mHealth text messaging**	**16**
Direct feedback	2
Interview	1
Key performance indicator	4
Model	1
Questionnaire	7
Usage logs	1
**Result**	**122**

### 3.2. Standardized and self-defined questionnaires

There are a total of 36 questionnaires among the evaluation tools. These include 28 standardized questionnaires with defined scores and eight individually defined questionnaires created by the app providers. Table 2 shows all questionnaires according to frequencies that occurred at least twice. Of the 28 standardized questionnaires, the validated generic EQ-5D questionnaire for measuring quality of life (*n* = 6, 21%) occurred most frequently.

**Table 2:** Standardized questionnaires that were used most frequently.

Standardized questionnaires	Frequency of questionnaires
EQ-5D-3L/EQ-5D-5L/EQ-5D-VAS	6
The Morisky Medication Adherence Scale (MMAS-8)	5
International Physical Activity Questionnaire (IPAQ)	5
Self-Care of Heart Failure Index (SCHFI)	4
Minnesota Living with Heart Failure Questionnaire (MLHFQ)	4
Health-related quality of life (HRQL)	3
System Usability Scale (SUS)	3
Short-Form 36 (SF-36)	3
Hospital Anxiety and Depression Scale (HADS)	3
Patient Reported Outcomes Measurement Information System (PROMIS-10)	2
Patient Health Questionnaire (PHQ-8 item)/ (PHQ-9 item)	2
Result	40

Overall, the contents of the questionnaires most frequently related to the usability (*n* = 10, 28%) and quality of life (*n* = 8, 22%). Among the ten questionnaires for usability, 8 were self-defined questionnaires, and only 2 were standardized questionnaires. It could be additionally determined that about 17% (*n* = 6) of the questionnaires are focused on disease-specific content, especially chronic and heart diseases. To assess medication adherence and psychological well-being while using the app, only questionnaires were used, such as the Morisky Medication Adherence Scale (MMAS-8) (19, 26–28, 47) or the Hospital Anxiety and Depression Scale (HADS) (23, 45, 47). Physical activities have been measured using the International Physical Activity Questionnaire (IPAQ) (16, 21, 31, 43, 45) and the Godin Leisure Time Physical Activity Questionnaire (47). In addition, the study by *Beatty* et al. (14) conducted a semi-structured interview to assess physical activity. Technology use is the least studied among all the questionnaires (*n* = 1, 3%).

### 3.3. Use of technology and usability

Five assessment tools were identified from the 37 studies to assess technology usage. These included usage logs, semi-structured interviews, the Mobile Application Rating Scale (MARS), and two model constructs. The model constructs included the UTAUT2 and the Technology Acceptance Model. The goal of the UTAUT2 is to analyze the behavioral intention to use a telemonitoring system. Various factors are considered and evaluated using quantitative and qualitative research methods (52). Similarly, the technology acceptance model describes the extent to which a person believes their work performance can be enhanced using the system (53).

To evaluate usability (*n* = 22), mainly self-defined questionnaires (*n* = 8, 36%) and semi-structured interviews (*n* = 5, 23%) have been used. In addition, three studies (14, 21, 30) used the standardized questionnaires “System Usability Scale” and one study (34) used the Perceived Health Web Site Usability Questionnaire.

### 3.4. Economic measurements and key indicators

Of the 37 studies, 12 (13, 16, 22, 25, 27, 29, 31, 35–38, 40) used key indicators to evaluate the app. Table 3 shows the frequency of instruments that used key indicators (*n* = 22) and the associated dimension.

**Table 3:** Number of instruments used, and the associated dimension.

Dimensions and the associated key indicators	Instrument quantity
**Economic measure**	**21**
Hospital readmissions	9
Mortality	4
Length of stay (HF related and all cause)	2
Cost-effectiveness analyse	2
Health care costs using the guide of costs of the Ministry of Health, Social Policy and Equality	1
Number of visits to the ED (HF related and all cause)	1
Number of visits to the outpatient clinic	1
Incremental cost-effectiveness ratio (ICER)	1
**Quality of life**	**1**
Quality-adjusted life year (QALY)	1
**Result**	**22**

Apart from the quality-adjusted life year (QALY) measure, the evaluation tools are almost exclusively for economic measures. The QALY is a measure that puts the lifetime gained by a measure in relation to the quality of life present in this time interval (54). The vast majority paid particular attention to hospital readmissions (*n* = 9, 41%) and mortality (*n* = 4, 18%). A specific cost-effectiveness analysis was performed in a total of 2 studies (19, 28).

## 4. Discussion

Based on the analysis, it has become clear that questionnaires are among the most frequently used evaluation tools for mHealth in CVD, which is the case for all application types (mHealth app, mHealth system, mHealth text messing). Quantitative methods mean less time and cost for researchers due to validated and meaningful data and are a popular method. Questionnaires are mainly used to improve usability and assess changes in quality of life. It is noticeable that predominantly standardized questionnaires were used with which a dimension can be evaluated specifically. However, self-defined questionnaires were primarily used to evaluate usability instead of the specific standardized questionnaires, System Usability and Perceived Health Web Site Usability Questionnaire. Heiney et al. (22) reasoned as follows (…) *we were unable to identify an evaluation tool specifically for mHealth apps and this population*. *For example, the Systems Usability Scale was too broad to help us identify specific problems with the phone and the app. We asked closed questions that assessed potential problems with the phone and messages* (i.e.*, readability*) (…). The example shows that app providers do not yet use reliable, standardized evaluation tools to assess the usability of a mHealth application. Subjective questions, especially about usability and visual appeal, are challenging to standardize but are among the essential features that drive user engagement with apps (55). Open-ended feedback (33, 43, 48) or semi-structured interviews (14, 16, 19, 41, 49) are preferred to identify specific improvements to the app’s functionality.

Besides questionnaires, among the most widely used assessment tools are usage logs to evaluate user updates and adherence. Usage logs help track the patient’s interaction with the application. From this, patient motivation can be determined. Engagement and acceptance are essential to integrate mHealth interventions into care in a long-term and resource-efficient manner.

Comprehensive economic calculation bases occur only in 3 studies (16, 31, 40). The study by *Cano Martín* et al. (40) evaluates the economic impact of using a mobile app for the self-management of heart disease. To this end, a cost-effectiveness analysis was conducted. It is concluded that the app’s introduction could result in a 33% reduction in the cost of managing and treating the disease. From today’s perspective, savings like these are essential to cap limited resources and rising costs in healthcare systems. Another economic indicator for calculating the quality of life was examined in the study by *Sankaran* et al. (16). The improvement in quality of life by the mHealth application was measured using a QALY calculation. Calculations such as these provide the decisive impetus for health insurance companies to finance new forms of therapy.

Evaluation methods regarding data transfer between app providers and patients were not available. According to a survey (56), 45% of consumers expressed concerns related to the unintended use and sharing of personal health data. Therefore, to reduce consumer concerns, it is recommended to ensure a transparent presentation of results by evaluating a privacy system, e.g., “Privacy Management Platform.”

### 4.1. Limitations

Due to the scope of this study, we focused on individual-level instruments used to evaluate CVD mHealth applications. Individual medical measurements, such as various laboratory tests or vital sign measurements, were not considered due to the diversity and multiplicity of these indicators due to the heterogeneity of the applications included in the study. Nevertheless, in further studies, we would like to investigate the research need for methods to evaluate clinical endpoints for CVD apps. In addition, in a further study we plan to analyze the effects reported for each mHealth app among the studies with the aim of investigating to what extent the different types of evaluation do or do not lead to different ranges of effects.

### 4.2. Conclusion

This study aimed to analyze evaluation tools from 37 studies applied to CVD mHealth interventions. The resulting data shows that quantitative questionnaires, use logs, or key indicators are predominantly used. Regarding the questionnaires, currently, there are few standardized questionnaires to determine usability for mHealth applications. Here, a need for research arises for developing new or optimizing existing questionnaires. Existing theoretical constructs such as the UTAUT2 or the technology acceptance model, which can assess more in-depth individual use of the technology, are only used in a few studies. However, these should not be disregarded as such guideline mHealth interventions are used to optimize and improve for patients. A comprehensive framework that identifies and operationalizes the criteria for assessing mHealth applications can provide long-term policy recommendations on the impact of mHealth applications and is, therefore, essential for the further development of the healthcare system.

For this reason, economic metrics are also an essential consideration for evaluating mHealth applications. More profound calculations, such as cost-effectiveness analysis or QALY, should be included in an evaluation framework in addition to hospital KPIs (mortality, readmission, etc.) so that new forms of therapy, such as mHealth applications, can be reimbursed by health insurers in the future. mHealth apps have great potential to improve the quality and efficiency of services. To ensure quality, testing mHealth applications for their objective benefits is important.

## Data availability statement

The original contributions presented in the study are included in the article/Supplementary material, further inquiries can be directed to the corresponding author.

## Author contributions

JK, WS, and FH contributed to the conception and design of the study and wrote sections of the manuscript. JK performed the data analysis and wrote the first draft of the manuscript. All authors contributed to the article and approved the submitted version.

## Conflict of interest

The authors declare that the research was conducted in the absence of any commercial or financial relationships that could be construed as a potential conflict of interest.

## Publisher’s note

All claims expressed in this article are solely those of the authors and do not necessarily represent those of their affiliated organizations, or those of the publisher, the editors and the reviewers. Any product that may be evaluated in this article, or claim that may be made by its manufacturer, is not guaranteed or endorsed by the publisher.
