# Supplementary material for: Examining standardized tools used for the evaluation of mobile health applications for cardiovascular disease
Source: Front Public Health. 2023 Jun 14;11:1155433. doi: 10.3389/fpubh.2023.1155433 (PMC10303135; doi:10.3389/fpubh.2023.1155433)
Supplement: Supplementary file 1 [file Table_1.docx]

Supplementary material 4: Extracted information of the included studies

| **ID** | **Title** | **Authros** | **Year** | **Journal** | **Link/DOI** | **Country** | **Type of Intervention** | **Study design** | **Evaluation methods qualitative and quantitative** | **Evaluations instruments** |
| --- | --- | --- | --- | --- | --- | --- | --- | --- | --- | --- |
| 8 | Mobile Phone-Based Telemonitoring for Heart Failure Management: A Randomized Controlled Trial | E. Seto, K. J. Leonard, J. A. Cafazzo, J. Barnsley, C. Masino, H. J. Ross | 2012 | Journal of Medical Internet Research | DOI: 10.2196/jmir.1909 | Canada | mHealth System - Telemonitoring   Devices: mobile phone, weight scale, blood pressure monitor, ECG recordings  Interfacing Tool Documentation (on health data)  Clinician Tool (Alerts) | RCT  Sample: N=100  Duration: 6 months  Retention rate: 94% Loss to follow-up: 6 | Standarized questionnaires Collection of hospital KPIs data | MLHFQ SCHFI Hospital readmissions, number of nights in hospital, and mortality |
| 9 | VA FitHeart, a Mobile App for Cardiac Rehabilitation: Usability Study. | A. Beatty, S. L. Magnusson, J. C. Fortney, G.G. Sayre, M. A. Whooley | 2018 | JMIR Human Factors | DOI: 10.2196/humanfactors.8017 | United States of America | mHealth App "VA FitHeart" - CR  Self-Monitoring Tool  Documentation on health data (bp, pulse, weight, glucose, cholesterol) Reminder Feedback | Usability Study  Sample: N=15 Duration: - Retention rate: 87% Loss to follow-up: 2 | Interviews Standarized questionnaires  Usability testing Guidance by UTAUT2 construct | SUS UTAUT2 Semistructured Interviews; physical activity and the use of technology |
| 10 | Feasibility of a Smartphone-enabled Cardiac Rehabilitation Program in Male Veterans With Previous Clinical Evidence of Coronary Heart Disease. | A. Harzand, B.Witbrodt, M.L. Davis-Watts, A. Alrohaibani, D.Goese, N. K. Wenger, A. J. Shah, A. Maziar Zafari | 2018 | Am J Cardiol. | DOI: 10.1016/j.amjcard.2018.07.028 | United States of America | mHealth System - CR Program   Devices: App, Monitoring Dashboard  Interfacing Tool  Reminder Education (Videos, Exercising) Documentation  Training Tool | Single-arm prospective study Sample: N=18 Duration: 3 months Retention rate: 72% Loss to follow-up: 5 | Semi-qualitative survey Usage logs | Usage logs; engagement Semi-qualitative survey; acceptability |
| 11 | Evaluating the Impact of the HeartHab App on Motivation, Physical Activity, Quality of Life, and Risk Factors of Coronary Artery Disease Patients: Multidisciplinary Crossover Study. | S. Sankaran, P. Dendale, K. Coninx | 2019 | JMIR Mhealth and Uhealth | DOI: 10.2196/10874 | Belgium | mHealth App "HeartHab App " - CR  Interfacing Tool Education (e-coaching, videos) Documentation Clinician Tool (Alerts) Reminder (Exercises) | Mixed-Methods Study Sample: N=32 Duration: 4 months Retention rate: 88% Loss to follow-up: 4 | Semistructured interviews Standarized questionnaires  Usage logs | IPAQ HeartQoL EQ-5D-5 Interviews; Experience with technology, medication, lifestyle QALYs Usage logs; all interactions with the app |
| 12 | Mobile Health Technology for Atrial Fibrillation Management Integrating Decision Support, Education, and Patient Involvement: mAF App Trial | Y. Guo,Y. Chen, D. A. Lane, L. Liu, Y. Wang, G. Y.H. Lip | 2017 | The American Journal of Medicine | DOI: 10.1016/j.amjmed.2017.07.003 | China | mHealth App "mFA"   Self-Management Tool Education Documentation Clinical Decision Support Reminder (Medication) | Cluster randomized trial Sample: N=209 Duration: 3 months Retention rate: 80% Loss to follow-up: 42 | Standarized questionnaires | EQ-5D-Y Atrial fibrillation knowledge scale Pharmacy Quality Alliance adherence measure Satisfaction rate; usability, feasibility, and acceptability ACTS 3-item Adherence Estimator scores |
| 13 | Evaluating the Utility of mHealth ECG Heart Monitoring for the Detection and Management of Atrial Fibrillation in Clinical Practice. | K. T. Hickey, A. B. Biviano, H. Garan, R. R. Sciacca, T. Riga, K. Warren, A. P. Frulla, N. R. Hauser, D. Y. Wang, William Whang | 2017 | Journal of Atrial Fibrillation | DOI: 10.4022/jafib.1546 | United States of America | mHealth System ECG "AliveCorTM"  Devices: wireless ECG, App  Interfacing Tool Documentation Clinician Tool | Cohort study Sample: N=46 Duration: 6 months Retention rate: 76% Loss to follow-up: 11 | Standarized questionnaires | SF-36v2TM multi-item scale |
| 14 | Evaluating the Use of Mobile Health Technology in Older Adults With Heart Failure: Mixed-Methods Study. | L. L. Lefler, S. J. Rhoads, M. Harris, A. E. Funderburg, S. A. Lubin, I. D. Martel, J. L. Faulkner, J. L. Rooker, D. K. Bell, H. Marshall, C. J. Beverly | 2018 | JMIR Aging | DOI: 10.2196/12178 | United States of America | mHealth System   Devices: tablet, bluetooth-weight scale, Pulse wave blood pressure wrist monitor  Interfacing Tool Documentation Clinical Decision Support (Symptoms) Clinician Tool (Alerts) | Mixed-Methods Study Sample: N=28 Duration: 3 months Retention rate: 89%  Loss to follow-up: 3 | Standarized questionnaires Semistructured interviews | Psychological Empowerment Scale Krantz Health Opinion Survey MMAS-8 Technology Acceptance Model SUS SSQ-HF |
| 15 | Randomized controlled feasibility trial of two telemedicine medication reminder systems for older adults with heart failure | C. M. Goldstein, E. C. Gathright, M. A. Dolansky, J. Gunstad, A. Sterns, J. D. Redle, R. Josephson, J.W. Hughes | 2014 | J Telemed Telecare | DOI:10.1177/1357633X14541039 | United States of America | mHealth App  Self-Management Tool Reminder (Medication) Logs (medication) Education | RCT  Sample: N=60 Duration: one month Retention rate: 92% Loss to follow-up: 5 | Self-defined questionnaire Usage logs | Usage logs Questionnaires; Participants knowledge, satisfaction |
| 16 | The HEART Mobile Phone Trial: The Partial Mediating Effects of Self-Efficacy on Physical Activity among Cardiac Patients. | R. Maddison, L. Pfaeffli, R. Stewart, A. Kerr, Y. Jiang, J. Rawstorn, K. Carter, R. Whittaker | 2014 | Front Public Health | DOI: 10.3389/fpubh.2014.00056 | New Zealand | mHealth System - CR Program  Devices: mobile phone, device for internet support  Self-Management Tool Text messaging  Training Tool | RCT Sample: N=171 Duration: 6 months Retention rate: 92%  Loss to follow-up: 14 | Standarized questionnaires | IPAQ-LF |
| **17** | A Smartphone App for Self-Management of Heart Failure in Older African Americans: Feasibility and Usability Study | S. P. Heiney, S. B. Donevant, S. A. Adams, P. D. Parker, H. Chen, S. Levkoff | 2020 | JMIR Aging | DOI: 10.2196/17142 | United States of America | mHealth App "Healthy Heart"   Self-Management Tool  Education Documentation Clinical Decision Support (Symptoms) | Mixed-Methods Study Sample: N=12 Duration: one month Retention rate: 92% Loss to follow-up: 1 | Standarized questionnaires Collection of hospital KPIs data Self-defined questionnaire | HQOL14 SCHFI Questionnaire; potential problems with the phone and messages Hospital readmissions |
| **18** | A Behavioral Change Smartphone App and Program (ToDo-CR) to Decrease Sedentary Behavior in Cardiac Rehabilitation Participants: Prospective Feasibility Cohort Study. | N. Freene, S. van Berlo, M. McManus, T. Mair, R. Davey | 2020 | JMIR Formative Research | DOI: 10.2196/17359 | Australia | mhealth System "Vire" - CR Program  Devices: App, tracking Tools (accelerometer, wrist-worn Fitbit Flex), web-based program  Self-Management Tool  Reminder (exercise) Documentation  Training Tool (DO's) | Cohort Study Sample: N=21 Duration: 4 months Retention rate: 62% Loss to follow-up: 8 | Standarized questionnaires Usage logs Guidance by UTAUT2 construct | UTAUT2 MacNew Heart Disease Health-Related Quality of Life Questionnaire HADS |
| **19** | The Impact of Text Messaging on Medication Adherence and Exercise Among Postmyocardial Infarction Patients: Randomized Controlled Pilot Trial. | A. Pandey, A. A. Krumme, T. Patel, N. K. Choudhry | 2017 | JMIR Mhealth and Uhealth | DOI: 10.2196/mhealth.7144 | United States of America | mHealth - Text messaging  SMS-based Reminder (medication and excercise) Logs (medication) | RCT  Sample: N=84 Duration: 12 months Retention rate: 99% Loss to follow-up: 1 | Usage logs Self-defined questionnaire | Usage logs; exercise adherence Self-defined questionnaire; assess text message reminders |
| 20 | Impact on Readmission Reduction Among Heart Failure Patients Using Digital Health Monitoring: Feasibility and Adoptability Study. | C. Park C, E. Otobo, J. Ullman, J. Rogers, F. Fasihuddin, S. Garg, S. Kakkar, M. Goldstein, S. V.Chandrasekhar, S. Pinney, A. Atreja | 2019 | JMIR Medical Informatics | DOI: 10.2196/13353 | United States of America | mHealth System - Telemonitoring  Devices: Apps, blood pressure cuff, smart weight scale, web-based Dashboard, medicine software plattform  Interfacing Tool Documentation Clinician Tool (Alerts, Dashboard) | Registry study  Sample: N=60 Duration: one month Retention rate: 97% Loss to follow-up: 2 | Collection of hospital KPIs data Usage logs | Readmission rates Usage logs; drug adherence |
| 21 | Medication reminder applications to improve adherence in coronary heart disease: a randomised clinical trial | K. Santo, A. Singleton, K. Rogers, A. Thiagalingam, J. Chalmers, C. K. Chow, J. Redfern | 2019 | Heart (british cardiac society) | DOI: 10.1136/heartjnl-2018-313479 | Australia | mHealth Apps   Self-Management Tool Reminder (medication) | RCT  Sample: N=166 Duration: 3 months Retention rate: 92% Loss to follow-up: 14 | Standarized questionnaires Self-defined questionnaire | MMAS-8 item Questionnaire; accepatibilty, utility |
| 22 | Effect of a reminder system using an automated short message service on medication adherence following acute coronary syndrome | S. Khonsari, P. Subramanian, K. Chinna, L. A: Latif, L. W. Ling, O. Gholami | 2015 | European Journal of Cardiovascular Nursing | DOI: 10.1177/1474515114521910 | Malaysia | mHealth - Text messaging   Self-Management Tool  Reminder (medication) SMS-based | RCT  Sample: N=62 Duration: 2 months Retention rate: 97% Loss to follow-up: 2 | Standarized questionnaires Collection of hospital KPIs data | MMAS-8 item Hospital readmission and death rates |
| 23 | A text messaging intervention to promote medication adherence for patients with coronary heart disease: a randomized controlled trial | L. G. Park, J. Howie-Esquivel, M. L. Chung, K. Dracup | 2014 | Patient Education and Counseling | DOI: 10.1016/j.pec.2013.10.027 | United States of America | mHealth System - Telemonitoring  Devices: mobile phone, electronic pillbox, web-based platform  Self-Management Tool  Reminder (medication) Education SMS-based | RCT Sample: N=90  Duration: one month Retention rate: 93% Loss to follow-up: 6 | Standarized questionnaires  Usage logs | MEMS MMAS-8 |
| 24 | Patient engagement with a mobile web-based telemonitoring system for heart failure self-management: a pilot study. | S. Zan, S. Agboola, S. A. Moore, K. A. Parks, J. C. Kvedar, K. Jethwani | 2015 | JMIR Mhealth and Uhealth | DOI: 10.2196/mhealth.3789 | United States of America | mHealth System - Telemonitoring   Devices: Tablet, blood pressure cuff, weight scale, web-based platform, Interactive Voice Response system  Interfacing Tool  Self-Monitoring Documentation  Clinician Tool (Alerts, monitoring on platform) | Single-arm prospective study Sample: N=21 Duration: 3,2 months Retention rate: 95% Loss to follow-up: 1 | Self-defined questionnaire Usage logs Collection of hospital KPIs data Standarized questionnaires | PROMIS-10 MLHFQ PHQ-8 Hospital readmission Usage logs; web portal log-in |
| 25 | Feasibility of a Mobile Phone App to Promote Adherence to a Heart-Healthy Lifestyle: Single-Arm Study. | P. Lunde, B. B. Nilsson, A. Bergland, A. Bye | 2019 | JMIR Formative Research | DOI: 10.2196/12679 | Norway | mHealth App - CR program  Interfacing Tool  Education (behaviour change) Clinician Tool (Monitoring, Feedback/ instructions) Training | Single-arm prospective study Sample: N=14 Duration: 3 months Retention rate: 100% Loss to follow-up: 0 | Standarized questionnaires Usage logs Collection of hospital KPIs data | SUS Usage logs; adherence  Questionnaire; Patient experiences, open-ended questions SF-36 COOP/WONCA EQ-5D-VAS |
| 26 | A mobile phone intervention increases physical activity in people with cardiovascular disease: Results from the HEART randomized controlled trial | R. Maddison, L. Pfaeffli, R. Whittaker, R. Stewart, A. Kerr, Y. Jiang, G. Kira, W. Leung, L. Dalleck, K. Carter, J. Rawstorn | 2015 | European Journal of Preventive Cardiology | DOI: 10.1177/2047487314535076 | New Zealand | mHealth System - CR  Devices: mobile phone, web-based platform   Self-Management Tool  SMS-based & web-based Reminder (excercise) Education Training | RCT Sample: N=171 Duration: 6 months Retention rate: 89% Loss to follow-up: 18 | Standarized questionnaires Economic measurements | IPAQ-LF EQ-5D SF-36 Cost-effectiveness analyses |
| **27** | Long-term follow-up with a smartphone application improves exercise capacity post cardiac rehabilitation: A randomized controlled trial. | P. Lunde, A. Bye, A. Bergland, J. Grimsmo, E. Jarstad, B. B. Nilsson | 2020 | European Journal of Preventive Cardiology | DOI: 10.1177/2047487320905717 | Norway | mHealth App - CR  Interfacing Tool Reminder (excercise) Training Clinician Tool (monitoring, feedback) | RCT  Sample: N= 113 Duration: 12 months Retention rate: 98% Loss to follow-up: 2 | Standarized questionnaires | EQ-5D  HRQL |
| **28** | Effect of motivational mobile phone short message service on aspirin adherence after coronary stenting for acute coronary syndrome | J. Quilici, L. Fugon, S. Beguin, P. E. Morange, J. L. Bonnet, M. C. Alessi, P. Carrieri, T. Cuisset | 2013 | International Journal of Cardiology | DOI: 10.1016/j.ijcard.2013.01.252 | France | mHealth - Text messaging   Self-Management Tool Reminder (medication) SMS-based | RCT  Sample: N=521  Duration: one month Retention rate: 96% Loss to follow-up: 22 | Direct feedback | Feedback; drug adherence |
| **29** | A Hospital-Community-Family-Based Telehealth Program for Patients With Chronic Heart Failure: Single-Arm, Prospective Feasibility Study. | X. Guo, X. Gu, J. Jiang, H. Li, R. Duan, Y. Zhang, L. Sun, Z. Bao, J. Shen, F. Chen | 2019 | JMIR Mhealth and Uhealth | DOI: 10.2196/13229 | China | mHealth System - Telemonitoring  Devices: Apps, smart tracking devices (bp cuff, weight scale, wearable ECG), remote monitoring service platform  Interfacing Tool  Documentation  Clinician Tool (Monitoring, Feedback, Alerts) Education Self-Monitoring Electronic medical record Interaction by Text messages or video calls Reminder (tracking visits) | Single-arm prospective study  Sample: N= 70 Duration: 4 months Retention rate: 94% Loss to follow-up: 4 | Interviews Standarized questionnaires Self-defined questionnaire Usage logs | 12-item Perceived Health Web Site Usability Questionnaire Questionnaire; satisfaction  Interviews; lifestyle and health behaviors  Usage logs; engagement |
| 30 | Effect of Smartphone-Enabled Health Monitoring Devices vs Regular Follow-up on Blood Pressure Control Among Patients After Myocardial Infarction: A Randomized Clinical Trial. | R. W. Treskes, L. van Winden, N. van Keulen, E. T. van der Velde, S. Beeres;, D. E. Atsma, M. Schalij | 2020 | JAMA Network Open | DOI: 10.1001/jamanetworkopen.2020.2165 | Netherlands | mHealth System - Telemonitoring  Devices: App, weight scale, blood pressure monitor, rhythm monitor, step counter  Interfacing Tool Documentation  Electronic medical record Clinician Tool (Monitoring) | RCT Sample: N=200  Duration: - Retention rate: 90% Loss to follow-up: 20 | Standarized questionnaires Collection of hospital KPIs data | Patient’s Satisfaction Questionnaire; Satisfaction Self-defined Questionnaire; Patient’s Acceptance and Measurement Adherence  Mortality and hospital readmission |
| 31 | Outcomes of a Heart Failure Telemonitoring Program Implemented as the Standard of Care in an Outpatient Heart Function Clinic: Pretest-Posttest Pragmatic Study. | P. Ware, H. J. Ross, J. A. Cafazzo, C. Boodoo, M Munnery, E. Seto | 2020 | Journal of Medical Internet Research | DOI: 10.2196/16538 | Canada | mHealth System - Telemonitoring  Devices: App, weight scales, blood pressure monitors  Interfacing Tool  Documentation  Reminder (daily measurement) Clinician Tool (Alerts, Monitoring, Dashboard) | Single-arm prospective study Sample: N=315  Duration: 6 months Retention rate: 90% Loss to follow-up: 30 | Standarized questionnaires Collection of hospital KPIs data | Hospital readmission, number of all-cause hospitalizations, number of visits to the ED (HF related and all cause), length of stay (HF related and all cause), and number of visits to the outpatient clinic MLHFQ SCHFI EQ-5D-5L |
| 32 | Feasibility and Acceptability of Utilizing a Smartphone Based Application to Monitor Outpatient Discharge Instruction Compliance in Cardiac Disease Patients around Discharge from Hospitalization | A. M. Layton, J. Whitworth, J. Peacock, M. N. Bartels, P. Jellen, B. M. Thomashow | 2014 | International Journal of Telemedicine and Applications | DOI: 10.1155/2014/415868 | United States of America | mHealth App - CR  Interfacing Tool  Documentation  Reminder (medication, appointment, excercise) Education (parameter hier beschrieben) Clinician Tool (Alerts, Monitoring, Dashboard) | Qualitative Study  Sample: N=16 Duration: 2,2 months Retention rate: 25% Loss to follow-up: 12 | Usage logs Collection of hospital KPIs data | Readmission rates, length of stay Usage logs; acceptability and feasbility |
| 33 | Post-discharge short message service improves short-term clinical outcome and self-care behaviour in chronic heart failure | C. Chen, X. Li, L. Sun, S. Cao, Y. Kang, L. Hong, Y. Liang, G. You, Q. Zhang | 2019 | ESC Heart Failure | DOI: 10.1002/ehf2.12380 | China | mHealth - Text messaging   Self-Management Tool SMS-based  Reminder (medication, measurements) Education (f.e.x from Heart Failure Society of America, guidelines) | RCT  Sample: N=767  Duration: 6.4 months Retention rate: 95% Loss to follow-up: 37 | Collection of Hospital KPIs data Standarized questionnaires | Hospital readmission and mortality MLHFQ |
| 34 | Psychosocial factors and medication adherence among patients with coronary heart disease: A text messaging intervention | L. G. Park, J. Howie-Esquivel, M. A. Whooley, K. Dracup | 2015 | European Journal of Cardiovascular Nursing | DOI: 10.1177/1474515114537024 | United States of America | mHealth System - Telemonitoring  Devices: mobile phone, electronic pillbox  Self-Management Tool  Reminder (medication) Education SMS-based | RCT  Sample: N=90 Duration: one month Retention rate: 93% Loss to follow-up: 6 | Standarized questionnaires Usage logs | SEAMS Usage logs; drug adherence |
| 35 | Economic Impact Assessment from the Use of a Mobile App for the Self-management of Heart Diseases by Patients with Heart Failure in a Spanish Region | J. A. Cano Martín, B. Martínez-pérez, I. de la Torre-Díez, M. López-coronado | 2014 | Journal of Medical Systems | DOI: 10.1007/s10916-014-0096-z | Spain | mHealth App "CardiManager"  Self-Management Tool Education Reminder (medication, alert, entries) Documentation | RCT Sample: N=630  Duration: - Retention rate: 86% Loss to follow-up: 86 | Economic measurements | Cost-effectiveness analyses  Health care costs using the guide of costs of the Ministry of Health, Social Policy and EqualityCost-utility analysis using the incremental cost-effectiveness ratio (ICER) |
| 36 | Patients' Experiences of Using a Consumer mHealth App for Self-Management of Heart Failure: Mixed-Methods Study. | L. S. Woods, J. Duff, E. Roehrer, K. Walker, E. Cummings | 2019 | JMIR Human Factors | DOI: 10.2196/13009 | Australia | mHealth App - "Care4myHeart"  Self-Management Tool Documentation (weight) Reminder (medication, appointment) Education Clinical Decision Support (Symptoms) | Mixed-Methods Study Sample: N=8  Duration: 2 weeks < one month Retention rate: 75% Loss to follow-up: 2 | Standarized questionnaires  Semistructured Interviews | MARS Interviews; usability, feasibility |
| **37** | Patient Adherence to a Mobile Phone-Based Heart Failure Telemonitoring Program: A Longitudinal Mixed-Methods Study. | P. Ware, M. Dorai, H. J. Ross, J. A. Cafazzo, A. Laporte, C. Boodoo, E. Seto | 2019 | JMIR Mhealth and Uhealth | DOI: 10.2196/13259 | Canada | mHealth System - Telemonitoring  Devices: App, weight scales, blood pressure monitors  Interfacing Tool  Documentation  Reminder (daily measurement) Clinician Tool (Alerts, Monitoring, Dashboard) | Mixed-Methods Study Sample: N=231  Duration: 12 months Retention rate: 87% Loss to follow-up: 30 | Guidance by UTAUT2 construct Semistructured interviews Usage logs Self-defined questionnaire | Interwies; app adherence and experience UTAUT2 Usage logs; adherence Questionnaires, adherence |
| **38** | Using Mobile Health Intervention to Improve Secondary Prevention of Coronary Heart Diseases in China: Mixed-Methods Feasibility Study. | S. Chen, E. Gong, D. S, Kazi, A. B. Gates, R. Bai, H. Fu, W. Peng, G. De La Cruz, L. Chen, X. Liu, Q. Su, N. Girerd, K. M. Karaye, K. F. Alhabib, L. L. Yan, J. D. Schwalm | 2018 | JMIR Mhealth and Uhealth | DOI: 10.2196/mhealth.7849 | China | mHealth - Text messaging  Interfacing Tool Text messages/ phone calls Reminder (medication) Clinician Tool (support prescription of evidence-based medicines) | Mixed-Methods Study Sample: N=190 Duration: 3 months Retention rate: 93%  Loss to follow-up: 13 | Standarized questionnaires Direct feedback | the 4-item Morisky Green Levine Scale IPAQ Survey; patient satisfaction, usability, acceptability, feedback |
| **39** | Feasibility, Safety, and Effectiveness of a Mobile Application in Cardiac Rehabilitation. | I. Nabutovsky, S. Ashri, A. Nachshon, R. Tesler, Y. Shapiro, E. Wright, B. Vadasz, A. Offer, L. Grosman-Rimon, R. Klempfner | 2020 | The Israel Medical Association Journal | PMID: 32558441 | Israel | mHealth System - CR Program  Devices: mobile phone, smart watch, monitoring system   Interfacing Tool Clinician Tool (Alerts, Monitoring, Coaching) Education Training Documentation Text messages | Single-arm prospective study Sample: N=22 Duration: 6 months Retention rate: 100% Loss to follow-up: 0 | Usage logs Standarized questionnaires | PHQ-9 PROMIS-10 Usage logs; amount of time |
| 40 | An Internet- and mobile-based tailored intervention to enhance maintenance of physical activity after cardiac rehabilitation: short-term results of a randomized controlled trial | K. Antypas, S. C. Wangberg | 2014 | Journal of Medical Internet Research | DOI: 10.2196/jmir.3132 | Norway | mHealth System - CR  Devices: mobile phone, web-based platform   Self-Management Tool Education Training Reminder (exercises) | RCT  Sample: N=69 Duration: 3 months Retention rate: 28% Loss to follow-up: 50 | Standarized questionnaires Usage logs | Usage logs; number of log-ins, time spent  IPAQ URICA-E2 scale PC-EX scale HADS |
| 41 | Evaluation of an mHealth-Based Adjunct to Outpatient Cardiac Rehabilitation | M. B. D. Rosario, N. H. Lovell, J. Fildes, K. Holgate, J. Yu, C. Ferry, G. Schreier, S. Y. Ooi, S. J. Redmond | 2018 | IEEE Journal of Biomedical and Health Informatics | DOI: 10.1109/JBHI.2017.2782209 | Australia | mHealth System - CR  Devices: app, blood pressure monitor, weight scale, web-based platform  Interfacing Tool  Clinician Tool (Dashboard, Monitoring) Text-messages Documentation Training | RCT  Sample: N=66 Duration: 6 months Retention rate: 77% Loss to follow-up: 15 | Standarized questionnaires | DASS-21 |
| 42 | Text Message and Internet Support for Coronary Heart Disease Self-Management: Results From the Text4Heart Randomized Controlled Trial. | L. Pfaeffli Dale, R. Whittaker, Y. Jiang, R. Stewart, A. Rolleston, R. Maddison | 2015 | Journal of Medical Internet Research | DOI: 10.2196/jmir.4944 | New Zealand | mHealth System "Text4Heart" - CR   Devices: mobile phone, web-based platform, pedometer  Self-management tool Reminder (medication) SMS-based Education/ lifestyle behaviour change | RCT Sample: N=123 Duration: 6 months Retention rate: 94% Loss to follow-up: 7 | Standarized questionnaires | AUDIT C Godin Leisure Time Physical Activity Questionnaire MMAS-8 Self-efficacy for Managing Chronic Disease 6-item scale the Brief Illness Perception Questionnaire HADS |
| 43 | Evaluating Reach, Acceptability, Utility, and Engagement with An App-Based Intervention to Improve Medication Adherence in Patients with Coronary Heart Disease in the MedApp-CHD Study: A Mixed-Methods Evaluation. | K. Santo, A. Singleton, C. K. Chow, J. Redfern | 2019 | Medical Sciences | DOI: 10.3390/medsci7060068 | Australia | mHealth Apps   Self-Management Tool Reminder (medication) | Mixed-Methods Study Sample: N=58 Duration: 3 months Retention rate: 26%  Loss to follow-up: 43 | Self-defined questionnaire Usage logs Direct feedback | Usage logs; login Feedback questionnaire; usefulness and ease-of-use Focus group discussion; usefulness, ease-of-use |
| 44 | A text messaging intervention to improve heart failure self-management after hospital discharge in a largely African-American population: before-after study | S. Nundy, R. r. Razi, J. J. Dick, B. Smith, A. Mayo, A. O'Connor, D. O. Meltzer | 2013 | Journal of Medical Internet Research | DOI: 10.2196/jmir.2317 | United States of America | mHealth - Text messaging  SMS-based Self-Management Tool Education Reminder (medication, appointment) | Single-arm prospective study  Sample: N=15  Duration: one month Retention rate: 40% Loss to follow-up: 9 | Usage logs Standarized questionnaires Semistructured interview | SCHFI Interviews; feasibility, usability |
