# Supplementary material for: Examining standardized tools used for the evaluation of mobile health applications for cardiovascular disease
Source: Front Public Health. 2023 Jun 14;11:1155433. doi: 10.3389/fpubh.2023.1155433 (PMC10303135; doi:10.3389/fpubh.2023.1155433)

Supplementary material 1:Inclusion and exclusion criteria.

| **PCC-Elements** | **Inclusion Criteria** | **Exclusion Criteria** |
| --- | --- | --- |
| Population | - Patients (> 18 years) with a diagnosed CHD (different diseases) - No limitation of the number of participants, origin, gender of the study participants | - Patients who are at risk of coronary heart disease - Relatives of cardiovascular patients e.g. children - Comorbid heart disease (e.g. congenital heart defect, heart transplant) - Healthy, voluntary study participants |
| Concept | *mHealth Application*     - Wearable mHealth applications for patients with CHD - Studies using qualitative or quantitative methods to evaluate mHealth applications (e.g. standardised questionnaires, quality guidelines, device data sets, usage logs) - No limitation of the evaluation parameters - Ready developed mHealth applications                       *Study design*     - Single study designs to evaluate an mHealth intervention for patients with CHD - English and German Literature | - mHealth applications for the use of exclusively: - Risk factors (e.g. high blood pressure) - Diabetes - Chronic Obstructive Pulmonary Disease - Pregnancy - Nutrition assessment (e.g. food tracking) - Sport and Wellness - Sensor technology (e.g. implanted sensor) - Applications that are only designed for health care providers e.g. Clinical Assessment Tool - Risk screening tool of CHD for the population - Pure descriptions of the apps (e.g. system, technical, programme, algorithm description) - Studies that evaluate only medical measurements        - Study protocols - Preliminary studies (e.g. for the development of the app) - Reviews (e.g. systematic reviews, scoping reviews) - Case Studys |
| Context | - No limitation of cultural parameters (e.g. geographical location, social origin, gender-specific interests) - No restriction of the setting, e.g. acute care, primary care, rehabilitation facilities - Full texts | - Unpublished literature |

Supplementary material 2: Search strategy.

| **Database** | **Search String** | **Databased**  **Specific filter** | **Date of Search** | **Results** |
| --- | --- | --- | --- | --- |
| PubMed | Heart Disease* OR Cardiovascular Disease* AND Mobile Health OR "MHealth" OR Smartphone App* AND Evaluation | Free Full Text  2000 to 2021 | 05.01.2021 | N = 2916 |
| Livivo | cardiovascular disease AND mHealth OR mobile health app AND evaluation | Free Full Text  2000 to 2020  Online  English | 13.01.2021 | N = 485 |
| ProQuest | (mHealth OR "mobile health" app) AND Evaluation AND cardiovascular disease | Free Full Text  2000 to 2021  English | 13.01.2021 | N = 1356 |
| **Hits accumulated in databases** | | | | N = 4757 |
| *+ Additional studies from reference lists of 37 Systematic Reviews* | | | | |
| PubMed |  |  | 06.04.2021 | N = 287 |
| **Total included in search** | | | | **N = 5044** |

Supplementary material 3: PRISMA Flow diagram.


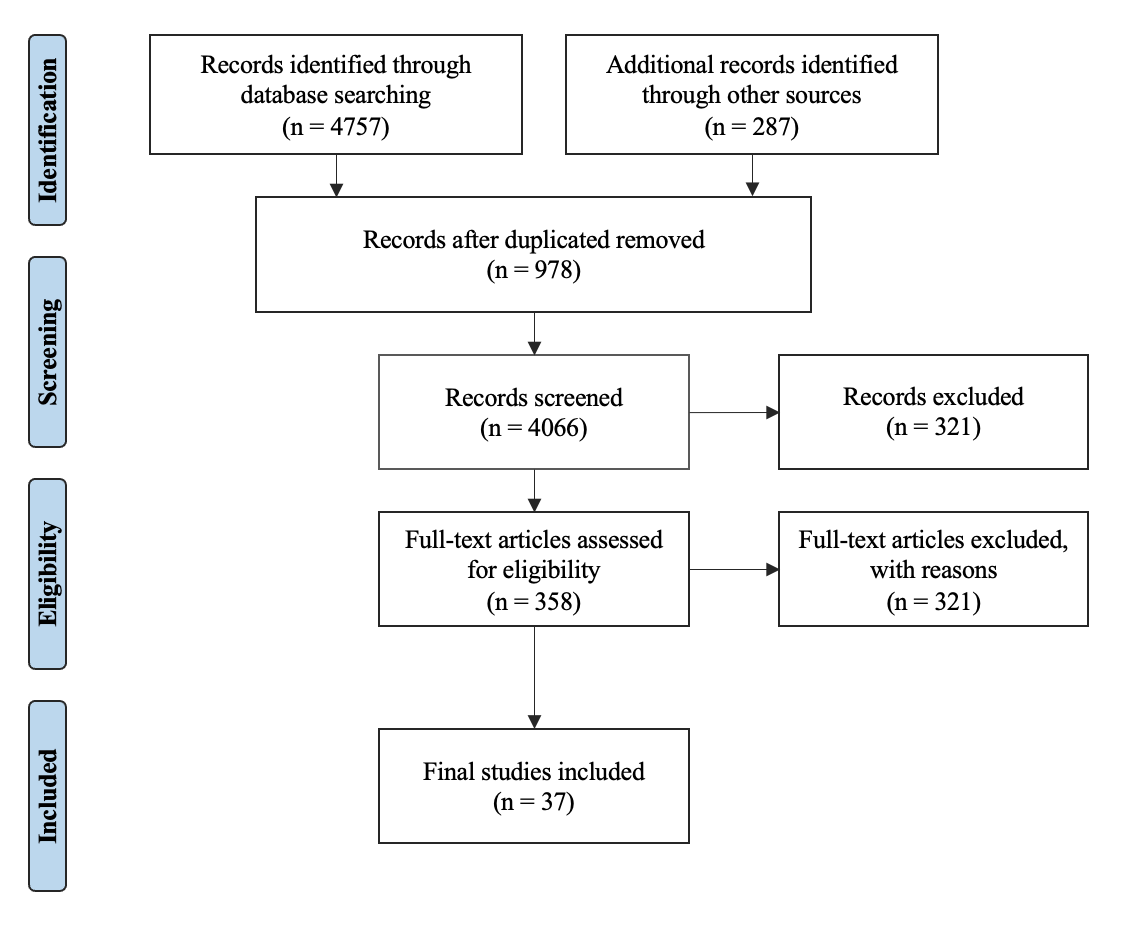

Supplement: Supplementary file 2 [file Data_Sheet_1.docx]
